# Supplementary figures and images for: Exploration of predictive factors based on oral and intestinal bacterial flora for treating patients with urothelial carcinoma
Source: PLoS One. 2025 Jun 18;20(6):e0324814. doi: 10.1371/journal.pone.0324814 (PMC12176287; doi:10.1371/journal.pone.0324814)

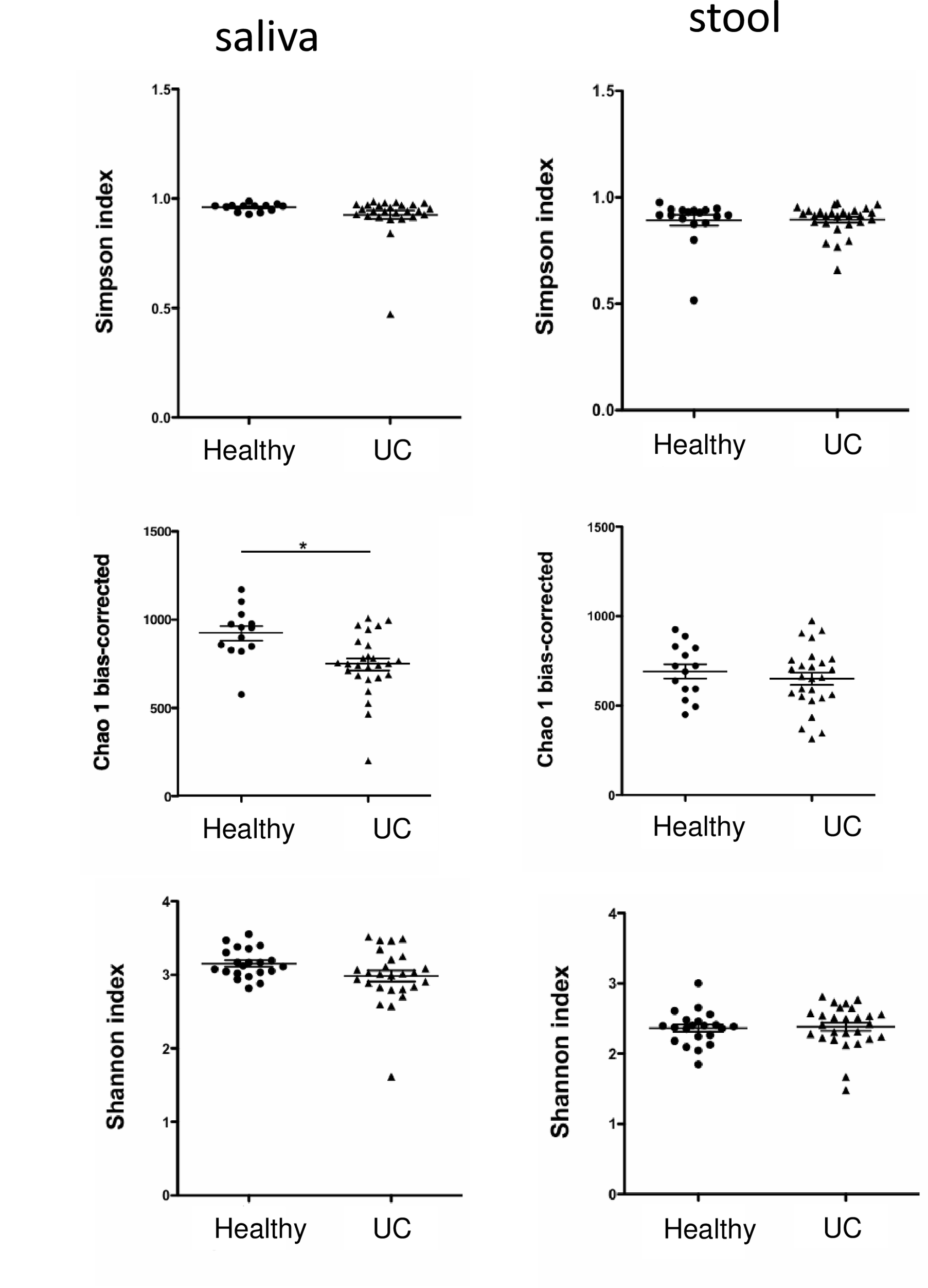

Supplement: S1 Fig — Detailed analysis of microbial composition across additional sample types or conditions. Specific comparisons and statistical comments are provided. (TIF) [file pone.0324814.s001.tif]

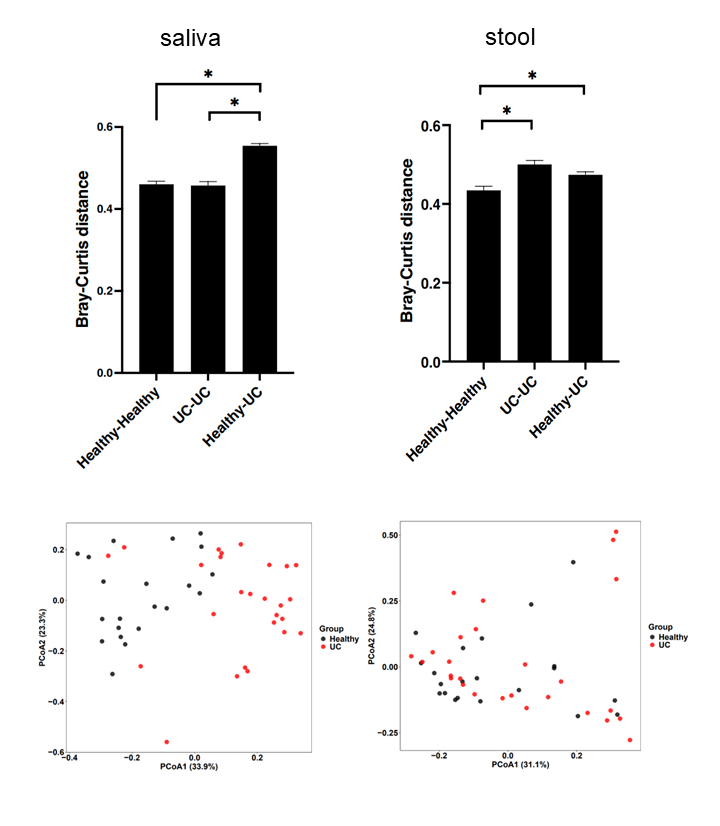

Supplement: S2 Fig — Illumina BaceSpace 16S For the count data of the families analysed by metagenomics, we used the vegan R package to calculate the Bray-Cruits distance and then performed intergroup comparisons and PCoA. For intergroup comparisons, we analysed the whole group using PERMANOVA. We also calculated and plotted the distances between Healthy, UC and Healthy-UC. We used the Wilcoxon test to test for significance in each group. (TIF) [file pone.0324814.s002.tif]
