# Supplementary material for: Exploration of predictive factors based on oral and intestinal bacterial flora for treating patients with urothelial carcinoma
Source: PLoS One. 2025 Jun 18;20(6):e0324814. doi: 10.1371/journal.pone.0324814 (PMC12176287; doi:10.1371/journal.pone.0324814)
Supplement: Attachment — Submitted filename: Cover letter rebuttal-20250411-clear.docx [file pone.0324814.s004.docx]

April 11^th^, 2025

Editor, *PLOS One*

Manuscript Number: PONE-D-25-02411

Title: Exploration of predictive factors based on oral and intestinal bacterial flora for treating patients with urothelial carcinoma

Dear Dr. Vinod Kumar Yata:

We are pleased to re-submit our revised manuscript for consideration for publication in *PLOS One.*

We are deeply grateful to you and the Reviewers for the constructive feedback provided on our initial submission. The comments and insights were very helpful and we have revised the manuscript in accordance with the Reviewers’ suggestions. Below, we present our point-by-point responses to these comments. Correspondingly, all revisions made to Manuscript are indicated in red for ease of review. We believe that these changes significantly enhance the quality of our work and hope that our revised manuscript now meets the esteemed standards for publication in your journal.

Best regards,

Yukihiro Hitaka

*********************************

Department of Urology

Yamaguchi University Graduate School of Medicine

1-1-1 Minami-Kogushi, Ube, Japan

Tel: +81-836-22-2275; Fax: -2276

Email: [h.yukki@yamaguchi-u.ac.jp](mailto:h.yukki@yamaguchi-u.ac.jp)

*********************************

****Point-by-point responses to the Reviewers’ comments****

**Reviewers’ comments:**

**Reviewer #1:**

Line 36: The abstract mentions using QIIME to generate operational taxonomic units, but OTUs are not mentioned anywhere in the manuscript. It appears all other analyses use web portal-based tools from Illumina; this discrepancy should be clarified.

Response:

Thank you for your comment. We did not use QIIME but Illumina’s web portal-based tool. We have corrected the sentence accordingly (lines 38–39 of the revised manuscript).

Line 158: The methods used for assessing compositional differences between groups are insufficiently described and supported here. My primary objection is in using the Student's t-test to evaluate differences in relative abundance—it's unclear whether relative abundance was tested using read counts, percentages, or some kind of transformed count, but figure 2 suggests the test was done on percentages. I am very skeptical that this is an appropriate method to use, as percentages within a sample are not independent: if one taxon grows significantly, the relative abundance of at least one other taxon, and likely many others, will fall. There are dozens of relevant papers debating the appropriate methods for differential abundance testing (e.g. https://doi.org/10.3389/fmicb.2017.02224); if the authors prefer to use the Student's t-test on relative abundances, I strongly believe it necessary that they provide references for why this is appropriate.

Response:

Thank you for your comments on the certification. We used the centred log-ratio (CLR) transformation to normalise the count data of saliva and stool from healthy individuals and UC family members. We have clarified this method in lines 177–181 of the revised manuscript.

160: This test is described in multiple places as the "non-parametric Student's t test"; the only Student's t-test I am aware of is certainly parametric.

Response:

Thank you for your comment. We have corrected the name of the test as “Mann-Whitney *U* test” (line 185 of the revised manuscript).

163: The manuscript states a significance threshold of 0.05, but does not specify how many tests were performed nor whether multiple test correction was applied. This is critical information: Figure 2 describes four tests covering two taxa, but presumably there were far more tests performed. If all families were tested for between-group differences, I would guess that even a generous approach to multiple test correction would alter most of the described p-values to be far above the set significance threshold. It is impossible for me to estimate any more accurately because this information was not available, but I strongly believe these results are invalid without a consideration of multiple testing and a description of how it was performed. This should be added.

Response:

Thank you for your comments on the certification. We have clarified that we performed multiple test correction using the Benjamini-Hochberg method in lines 181–184 of the revised manuscript.

I leave it to the editor to evaluate the authors' adherence to PLOS editorial policies, but I should note that the manuscript does not describe any deposition of sequencing data, and if any other data was submitted as supplementary files, I was unable to see them. As presented, the analysis described is impossible to replicate. I also am surprised to find that dozens of subjects were recruited, then samples were collected, prepped, sequenced and analyzed, without any disclosed funding.

Response:

Thank you for your comments on the certification. As suggested, we have described the deposition of sequencing in lines 187–188 of the revised manuscript. We have added our funding sources.

**Reviewer #2:**

Lines 81-83: Please define your specific aim more precisely.

Response:

Thank you for your comment. We have defined our specific aim more precisely in lines 85–89 of the revised manuscript.

Lines 86-88: The introduction's mention of Veillonellaceae and Prevotellaceae may suggest predetermined conclusions rather than objective investigation. Please reconsider this framing.

Response:

Thank you for pointing out the concern about the mention of the Veillonellaceae and Prevotellidae families. We have deleted this text as it lacks objectivity.

Lines 112 & 114: Please correct the sentence structure to eliminate repetition.

Response:

As pointed out, we corrected the structure of the sentence and eliminated the duplication.

Line 350: There is an inconsistency with Line 170. Figure 1 should present alpha diversity (Simpson and Shannon indices). Please correct this discrepancy.

Response:

Thank you for pointing out the discrepancy. We have corrected the figure number as Figure S1 (line 222 of the revised manuscript).

The methodology section lacks information on raw data processing. Please specify:

1) The number of reads per sample after processing

2) Whether samples were sequenced with adequate depth

3) Analysis of overall community differences (beta diversity)

4) Visualization of beta diversity through PCoA plots.

Response:

1) and 2) We have uploaded Excel files (saliva_reads_family.xlsx and stool_reads_family.xlsx) that specified the number of reads per sample and family after processing. These files show that samples were sequenced with adequate depth.

3) and 4) We have analysed the overall community differences and visualized the beta diversity through PCoA plots in Figure S2.
